# Morphometric Analysis of Radix Entomolaris: Implications for Endodontic Access and Treatment

**DOI:** 10.7759/cureus.59584

**Published:** 2024-05-03

**Authors:** Khyati Manik, Anuja Ikhar, Aditya Patel, Manoj Chandak, Jay Bhopatkar, Priyanka R Bhojwani, Shefali Singh

**Affiliations:** 1 Department of Conservative Dentistry and Endodontics, Sharad Pawar Dental College and Hospital, Datta Meghe Institute of Higher Education and Research, Wardha, IND; 2 Department of Orthodontics and Dentofacial Orthopaedics, Sharad Pawar Dental College and Hospital, Datta Meghe Institute of Higher Education and Research, Wardha, IND

**Keywords:** endodontics, radix entomolaris, mandibular molar, endodontic treatment, anatomical variation

## Abstract

Radix entomolaris (RE) is an anatomical variation that involves the existence of an extra root in lower molars, particularly the first molar. This variant, although less common, has significant clinical implications in endodontic treatment and dental surgeries. Appropriate detection of radix entomolaris is crucial for treatment planning and prognosis. Various diagnostic aids like radiography, cone-beam computed tomography, and intraoperative exploration aid in identifying this anatomical variant.

## Introduction

The primary goal of treating canals of the root is proper biomechanical preparation, and irrigation of root canals, followed by three-dimensional (3D) filling of an entire root canal, which inhibits the occurrence of pathology in the periapical region and improves the enhancement of the current endodontic expertise [1]. The majority of lower first molars have two roots, located mesially and distally [2]. The number and site of root and their canals in lower molars may vary. Carabelli introduced the radix entomolaris (RE), an extra third root in the case of lower molars [3].

According to various studies, the occurrence of RE is greater in the people of Taiwan (China), with a prevalence extending from 21.1% to 33.33% and incidence extending bilaterally from 53.65% to 68.57% [4]. Since RE occurs in these populations, it is probably a standard morphologic variant. Despite the greater occurrence of RE in many races, its incidence in the Indian populace is quite low, only 0.2% [5]. The exact etiology of RE is still not known but according to some authors, it may be due to disturbance during odontogenesis or may be due to the high degree of genetic penetrance [3].

Correct recognition and data of the atypical anatomy of the canals of root in addition to the location of the canal opening, chemical mechanical cleaning, and root canal shaping before filling the root canal with an airtight seal can all lead to the effective treatment of the root and its canals. The report focuses on diagnosis as well as effective treatment of RE in a lower first molar tooth while performing procedures like root canal therapy.

## Case presentation

**Figure 1:**
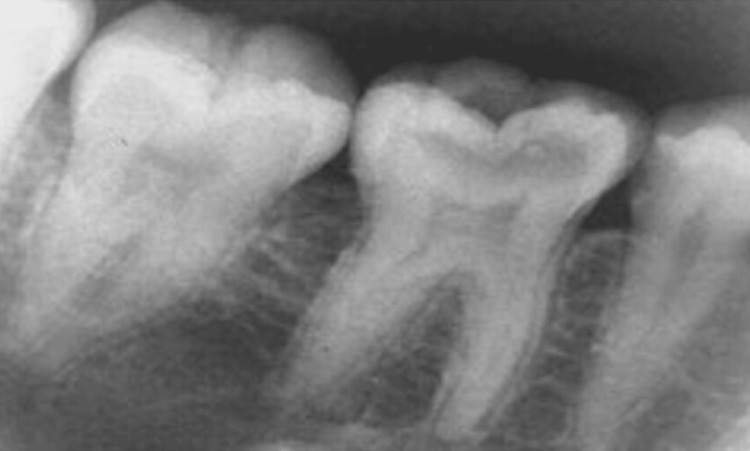
Preoperative radiograph

A 39-year-old male patient presented to the department of post-graduate endodontics with a chief complaint of pain in the lower right back of the jaw for a month. The pain was spontaneous in nature and persisted for minutes after the stimulus (usually heat, less often cold) was removed. The past medical history as well as past dental history of the patient was non-significant. Radiolucency of the enamel, dentin, and pulp, as well as periapical radiopacity with the right lower tooth, were detected on X-ray examination. The X-ray showed the presence of an accessory root located distally. The SLOB (Same Lingual Opposite Buccal) technique established the extra distolingual root (RE). As a result, a diagnosis of symptomatic irreversible pulpitis with apical periodontitis and condensing osteitis was made for the right lower first molar (Figure 1).

**Figure 2:**
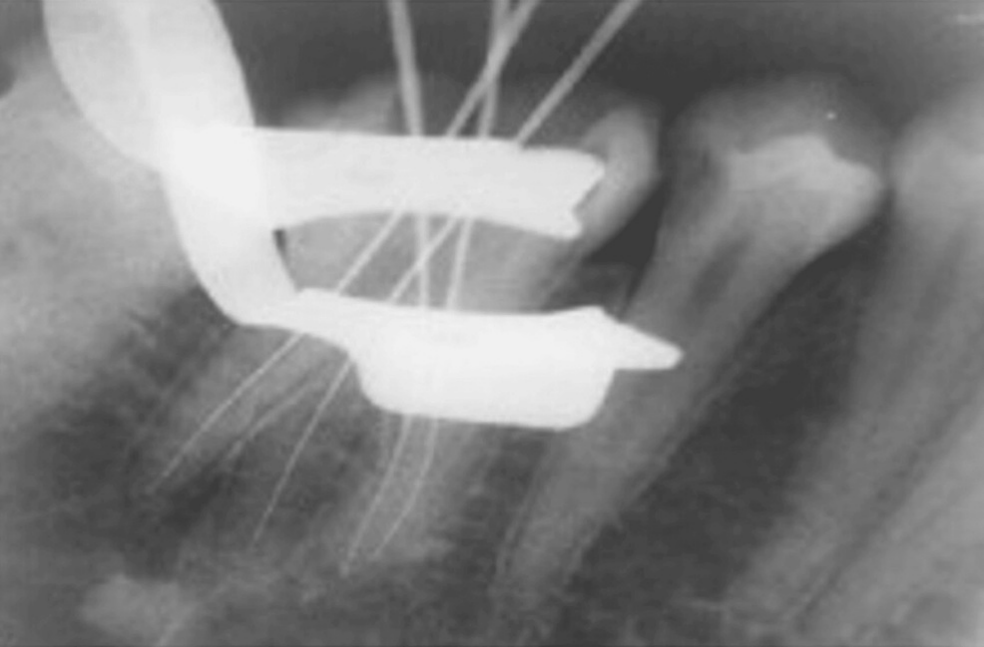
Working length radiograph

The patient received 2% xylocaine with 1:80,000 adrenaline. Rubber dam isolation was done. Round BR-45 (Mani Inc., Takenzawa, Japan) and Safe End bur EX-24 (Mani Inc.) were used to prepare the access cavity. After removing pulp tissue from the chamber, four openings were discovered. Working length was determined using Root ZX mini apex locator (J. Morita Corporation, Saitama, Japan) and verified by taking an angled radiograph (Figure 2).

Canals were irrigated and shaped using #25 rotary nickel-titanium (Ni-Ti) files (Guilin Woodpecker Medical Instrument Co., Ltd, Guilin, China) having a 6% taper. The canal was irrigated using 3% sodium hypochlorite (NaOCl) and 0.9% saline alternatively. Calcium hydroxide (RC Cal; Prime Dental Products Pvt Ltd, Thane, Maharastra, India) temporarily closed dressing (Neotemp; Orikam Healthcare India Pvt. Ltd, Gurgaon, Haryana, India) was applied and the patient was further called after one week.

**Figure 3:**
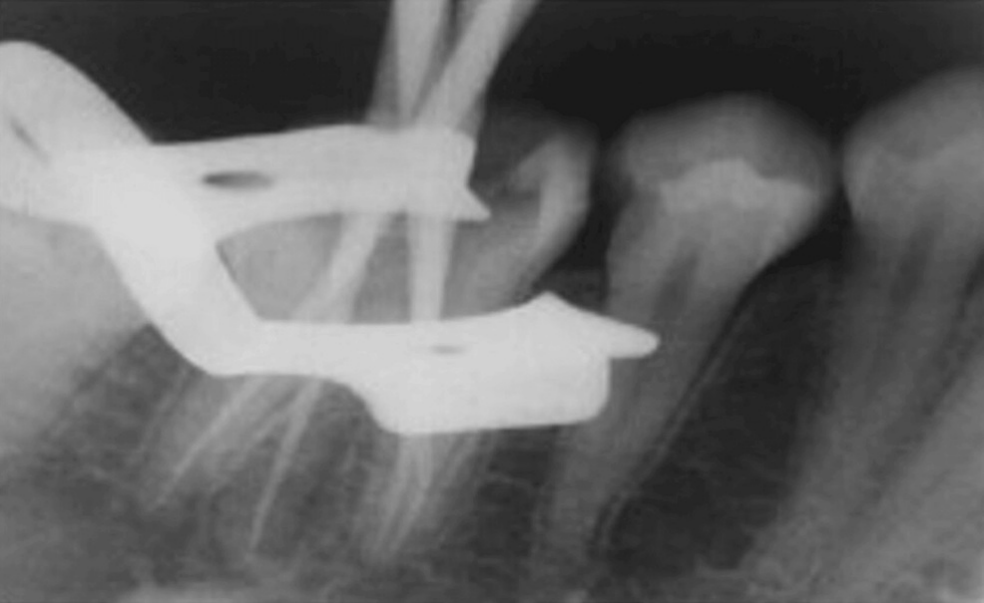
Master cone check

On the second appointment, the patient was completely free of symptoms. Provisional restoration was removed and all the canals were sonically activated with 17% ethylenediamine tetraacetic acid (EDTA) to facilitate calcium hydroxide removal. All the canals were then irrigated using 3% NaOCl and 0.9% saline alternatively. Gutta percha master cones were placed in the root canal and a radiograph was taken (Figure 3). 

**Figure 4:**
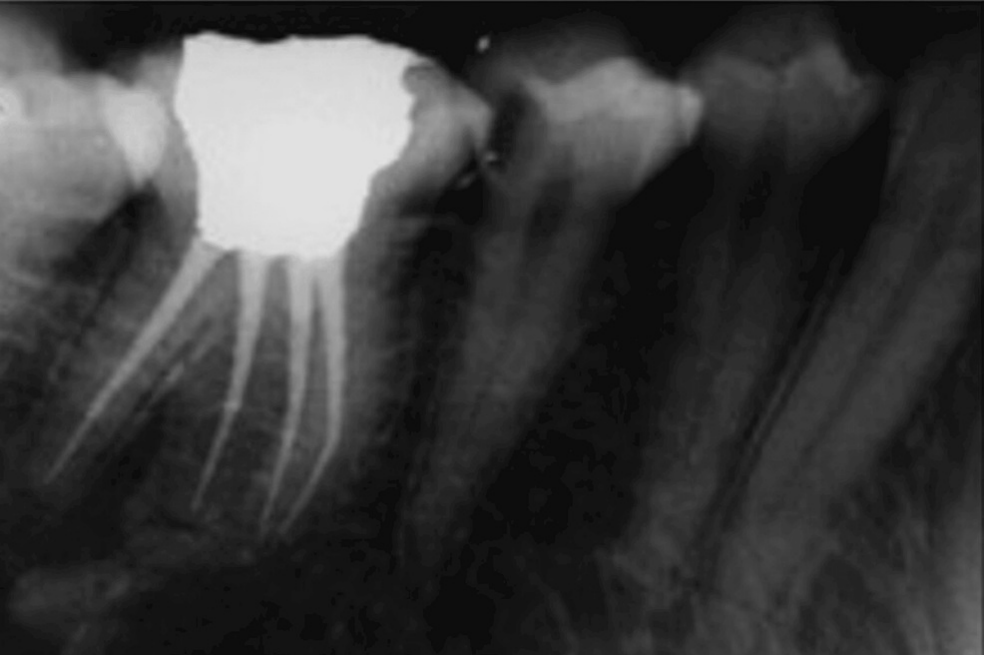
Post endodontic restoration

**Figure 5:**
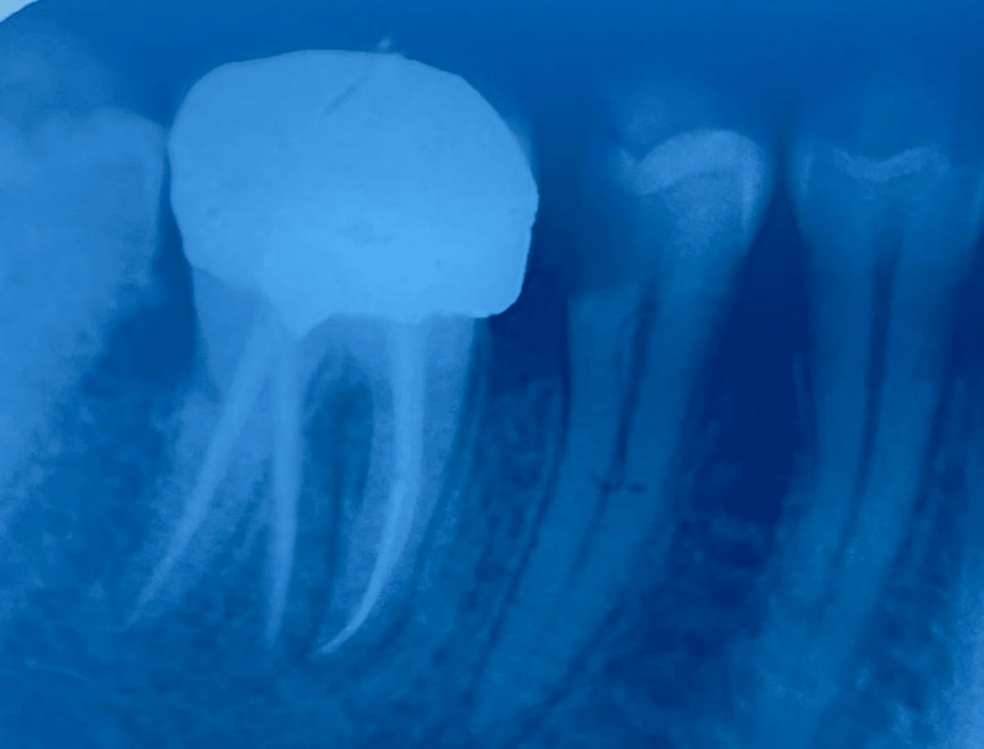
One-year follow-up image.

Obturation was carried out with master cones and epoxy resin-based sealer (Dia-ProSeal Root Canal Sealer; DiaDent Group International, South Korea). Post-endodontic composite restoration (Spectrum; Dentsply Sirona Inc., Charlotte, North Carolina, United States) was done (Figure 4). At the one-year follow-up, the patient was completely asymptomatic and there was no tenderness on percussion (Figure 5). 

## Discussion

The existence of a RE or a radix paramolaris has clinical consequences for endodontic treatment. A precise diagnosis of these additional roots can avert complications or a missed canal during root canal therapy. In the current case, the preoperative radiograph indicated that both roots of the RE overlap as it is usually located in a similar buccal-lingual plane of the “distobuccal root”. This was a remarkable finding. The RE orifice was placed distal to the direction of the mesiolingual from the foremost root canal present in the root distally. Triangle-shaped entrance cavity widened distolingually, resulting in a rectangle or trapezoidal presence. In case the entrance to the RE canal is not visible after removing the pulp chamber roof, it is necessary to inspect the floor and wall of this chamber more closely, particularly in the distolingual area.

Visual technology like dental loupes, dental operating microscopes, and cameras placed intraorally can be effective in these kinds of situations [6]. Clinically, with extensive knowledge of the laws of access cavity preparation, numerous procedures such as imagining canal bleeding points, dentinal map with the help of an explorer such as DG16, micro-openers for locating root canals, ultrasonic tips for toughening the grooves, 1% methylene blue dye for staining the floor of the pulp chamber, magnetic resonance microscopy, the champagne bubble test, and micro-CT are advantageous in locating the orifices of root canals [7].

Aside from radiographic diagnosis and analysis, clinical examination of the RE orifice while performing access opening is critical. The RE orifice is typically positioned distolingually from the foremost canal, and calcification is commonly present above the opening, which has to be removed for better-quality access and visibility [8]. Clinically, the existence of an additional tip of the cusp or more protruding lingual portion, together with prominent eminence and convexness of the cervical area, may show the existence of a supplementary root [9].

## Conclusions

Recognition of RE is crucial in various dental procedures, particularly endodontic treatment and surgical procedures involving the mandibular first molar. Failure to recognize and satisfactory care of the accessory root canal can lead to incomplete irrigation and biomechanical preparation of the canals of the root, potentially leading to procedure failure.
